# Histone demethylase RBP2 promotes malignant progression of gastric cancer through TGF-β1-(p-Smad3)-RBP2-E-cadherin-Smad3 feedback circuit

**DOI:** 10.18632/oncotarget.3756

**Published:** 2015-05-07

**Authors:** Xiuming Liang, Jiping Zeng, Lixiang Wang, Li Shen, Xueping Ma, Shuyan Li, Yujiao Wu, Lin Ma, Xinyu Ci, Qing Guo, Mutian Jia, Haiyu Shen, Yundong Sun, Zhifang Liu, Shili Liu, Wenjuan Li, Han Yu, Chunyan Chen, Jihui Jia

**Affiliations:** ^1^ Department of Microbiology/Key Laboratory for Experimental Teratology of Chinese Ministry of Education, School of Medicine, Shandong University, Jinan, PR China; ^2^ Department of Biochemistry, School of Medicine, Shandong University, Jinan, PR China; ^3^ Department of Pharmacology, School of Medicine, Shandong University, Jinan, PR China; ^4^ Department of Hematology, Qilu Hospital, Shandong University, Jinan, Shandong, PR China; ^5^ Department of Gastrointestinal Surgery, Jinan Central Hospital, Shandong University, Jinan, Shandong, PR China; ^6^ Department of Nursing, Ningxia Peoples Hospital, Yinchuan, PR China

**Keywords:** RBP2, TGF-β1, feedback circuit, metastasis

## Abstract

Some feedback pathways are critical in the process of tumor development or malignant progression. However the mechanisms through which these pathways are epigenetically regulated have not been fully elucidated. Here, we demonstrated that the histone demethylase RBP2 was crucial for TGF-β1-(p-Smad3)-RBP2-E-cadherin-Smad3 feedback circuit that was implicated in malignant progression of tumors and its knockdown significantly inhibited gastric cancer (GC) metastasis both *in vitro* and *in vivo*. Mechanistically, RBP2 can directly bind to E-cadherin promoter and suppress its expression, facilitating EMT and distant metastasis of GC. RBP2 can also be induced by TGF-β1, a key inducer of EMT, through phosphorylated Smad3 (p-Smad3) pathway in GC. The upregulated RBP2 can be recruited by p-smad3 to E-cadherin promoter and enhance its suppression, contributing to the promotion of metastasis of GC. In addition, the suppression of E-cadherin by RBP2 attenuated inhibition of Smad3 phosphorylation (exerted by E-cadherin), resulting further induction of RBP2 expression, and thus constituting positive feedback regulation during GC malignant progression. This TGF-β1-(p-Smad3)-RBP2- E-cadherin-Smad3 feedback circuit may be a novel mechanism for GC malignant progression and suppression of RBP2 expression may serve as a new strategy for the prevention of tumor distant metastasis.

## INTRODUCTION

GC malignant progression is the main reason for its poor prognosis, during which EMT (epithelial-to-mesenchymal transition) is critical. Characterized by downregulation of epithelial markers (e.g. E-cadherin and Occludin) and upregulation of mesenchymal markers (e.g. Vimentin, Snail-1, Slug, Twist and ZEB1), EMT enhances mobility of cancer cells, facilitating their distant metastasis [1–3]. The core event of EMT is the loss or inhibition of epithelial markers (such as E-cadherin) which causes damage to the conjunction between cells. In addition, tumor cells often obtain the stem cell property during EMT which is the reason why it is sometimes easy for cancer (including GC) to relapse [4]. In summary, EMT is accountable for tumor metastasis and leads to tumor relapse. Therefore it is urgent to unveil the underlying mechanism for EMT and this is instrumental in understanding the cause for tumor malignant progression.

It is well established that some signaling pathways are dominantly accountable for EMT as preceding reports have shown [5, 6]. TGF-β1, the most famous inducer of EMT, binds to its receptors on cell membrane and then, in most cases, phosphorylates Smad3. The phosphorylated Smad3 (p-smad3) binds to Smad4 (co-Smad) to form a complex and then will be translocated to the nucleus where it can promote or suppress downstream EMT-related genes expression [7, 8]. Other signaling pathways and many important transcriptional factors are involved in EMT [9, 10]. However, whether epigenetic molecules play a similar important role in EMT regulation and their potential relationship with established canonical signaling pathways essential for EMT is not fully understood. In addition, how transcriptional factors repress rather than activate expression of epithelial markers [11], such as E-cadherin and Occludin, in EMT process has not been thoroughly elucidated and epigenetic regulation may help to unveil the underlying mechanisms.

Moreover, mounting data indicate some feedback circuits are critical for tumor development and malignant progression [12, 13]. It was reported that perturbation of MicroRNA-370/Lin-28 homolog A/nuclear factor kappa B regulatory circuit contributes to the development of hepatocellular carcinoma [14]. It also remains unclear whether epigenetic molecules promote establishment of feedback circuits that contributes to tumor development and malignant progression.

Increasing investigation pay attention to epigenetic regulation during the process of tumor malignant progression [15]. Some histone demethylases have been proved to be crucial for tumor metastasis. It was published that KDM5B histone demethylase controlled epithelial-mesenchymal transition of cancer cells by regulating the expression of the microRNA-200 family [16], and Histone demethylase KDM6B promoted epithelial-mesenchymal transition [17]. Our group also found that JMJD2B promoted epithelial-mesenchymal transition by cooperating with β-catenin and enhanced gastric cancer metastasis [18]. We are now focusing on RBP2, the newly identified histone demethylase, which is closely associated with cancer development and progression [19–22]. Furthermore, some investigation have suggested the potential relationship between RBP2 and TGF-β1 as well as other canonical EMT-related pathways [23]. However, whether RBP2 appears to be crucial for malignant progression of human tumors and the underlying mechanisms have not been thoroughly investigated.

In our present work, we found the role RBP2 played in establishing feedback circuit that promoted malignant progression of GC. For the first time, we demonstrate that the TGF-β1-(p-Smad3)-RBP2-E-cadherin-Smad3 feedback circuit may be a novel mechanism for EMT and GC metastasis and targeting RBP2 expression may have therapeutic advantage for the prevention of tumor metastasis.

## RESULTS

### RBP2 expression was positively correlated with differentiation status and distant metastasis in primary gastric cancer tissues

**Figure 1:**
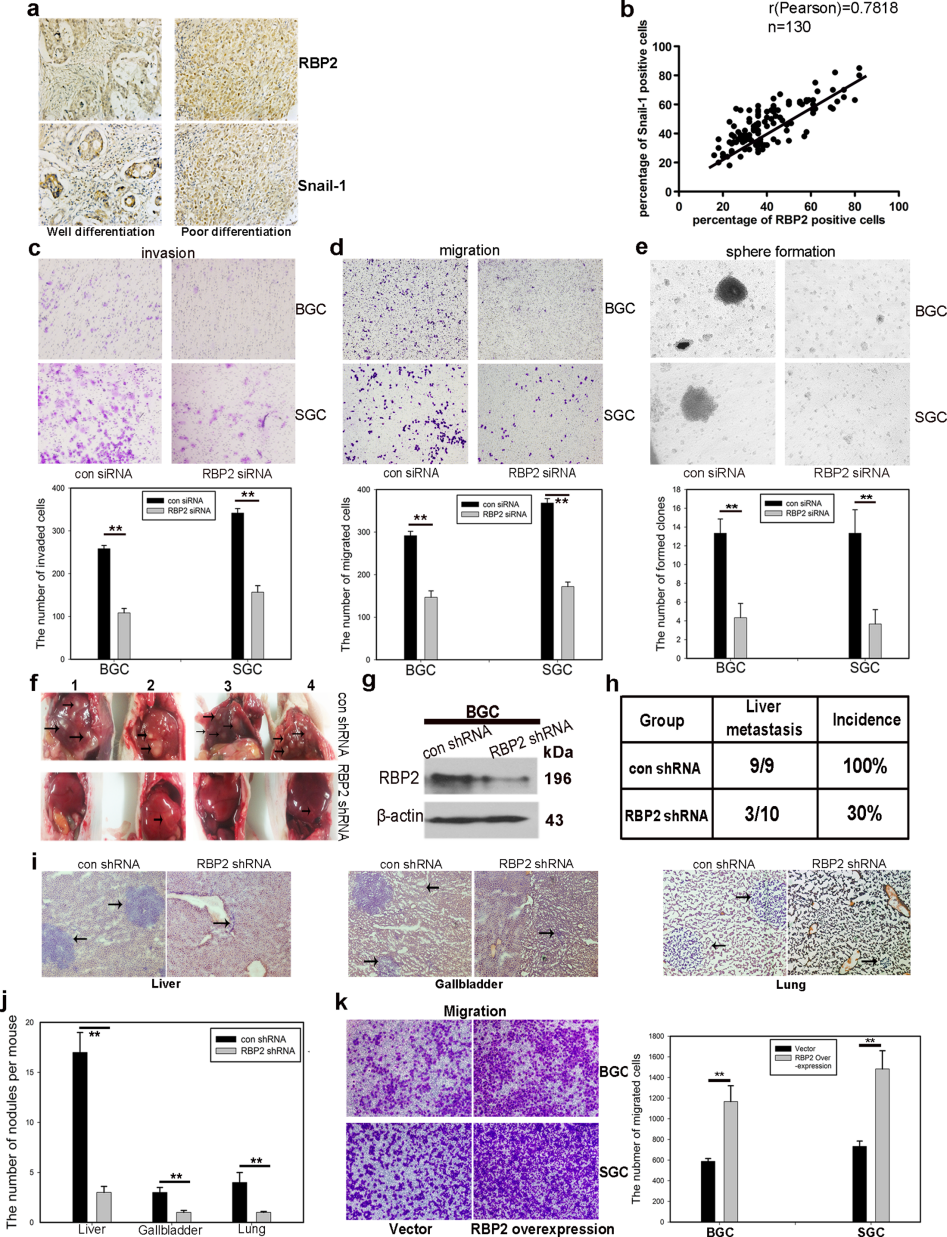
RBP2 expression was positively correlated with differentiation status and distant metastasis in primary gastric cancer tissues and it is involved in GC progression and GC stemness property maintenance **a.** Representative images of RBP2 and Snail-1 expression in differentiation status. **b.** RBP2 and Snail-1 expression was positively correlated in clinical specimen. **c and d.** Knockdown of RBP2 decreases cell invasion (c) and cell migration (d) Data are mean ± SD of 3 biological replicates, **p < 0.01 compared with negative control. Original magnification, × 40. **e.** Stem cell property is jeopardized with RBP2 inhibition. Data are mean±SD of 3 biological replicates, **p < 0.01 compared with negative control. Original magnification, × 40. **f.** RBP2 suppression significantly decreases liver metastasis. Black arrows indicate the metastatic tumor nodules. **g.** western blot indicates the inhibition of RBP2 in lenti-virus mediated stable-transfection BGC-823 cells. **h.** Decrease of liver metastasis incidence in RBP2 shRNA group. **i.** and **j.** HE staining of liver, gallbladder and lung, which shows decrease of metastatic tumor nodules formed in RBP2 shRNA group. Representative images are shown here. Black arrows indicate the metastatic tumor nodules. Original magnification, × 40. Data are mean ± SD of 3 biological replicates, **p < 0.01 compared with negative control. **k.** RBP2 overexpression enhanced migration of gastric cancer cells. Original magnification, × 40. Data are mean ± SD of 3 biological replicates, **p < 0.01 compared with negative control.

Our group has evidenced the high expression of RBP2 in primary gastric cancer tissues, however, whether RBP2 expression correlated with tumor progression remained unclear. We collected 130 cases of primary gastric cancer specimen and analyzed RBP2 expression there. As expected, we found the expression of RBP2 was correlated with differentiation status and distant metastasis in these tissues. Here, we also detected Snail-1, which was an established marker of tumor metastasis, whose expression was thought as a positive control. Expression of RBP2 and Snail-1 had no relationship with ages (Table 1, *p* > 0.25 and *p* > 0.5 respectively) and gender (Table 1, *p* > 0.25 and *p* > 0.5 respectively) of patients involved. However, both RBP2 and Snail-1 was higher expressed in poor differentiated tumors than in moderate or well differentiated ones (Figure 1a, Table 1, *p* < 0.005), and in tumors with distant lymphatic metastasis than in these without metastasis (Table 1, *p* < 0.005 and *p* < 0.025 respectively). Even in the same patient, the expression of RBP2 and Snail-1 in poor differentiated regions (Supplementary Figure 1a, Red arrows) was remarkably higher than that in well or moderate differentiated regions (Supplementary Figure 1a, Black arrows). Furthermore, we found RBP2 expression was positively correlated with the expression of Snail-1 in these tissues (Figure 1b, Table 1), implicating RBP2 may have a similar role to promote tumor progression as Snail-1 did.

**Table 1:** Correlation between RBP2 and Snail-1 expression and their expression relationship with clinicopathological features in primary gastric cancer specimen

Part I: RB P2 and Snail-1 expression relationship with clinicopathological features in primary gastric cancer specimen									
		RBP2 expression				Snail-1 expression			
Groups	Total	0––1	2––3	χ^2^ value	*p* value	0––1	2––3	χ^2^ value	*p* value
Age (years)									
<60	51	17(33.3%)	34(66.7%)	1.33	*p* > 0.25	5(9.8%)	46(90.2%)	0.17	*p* > 0.5
≥60	79	19(24.1%)	60(75.9%)			12(15.2%)	67(84.8%)		
Gender									
Male	104	31(29.8%)	73(70.2%)	0.97	*p* > 0.25	13(12.5%)	91(87.5%)	0.09	*p* > 0.5
Female	26	5(19.2%)	21(80.8%)			4(15.4%)	22(84.6%)		
Tumor Differentiation									
Well	28	22(78.6%)	6(21.4%)	66.71	*p* < 0.005	10(35.7%)	18(64.3%)	17.55	*p* < 0.005
Moderate	42	10(23.8%)	32(76.2%)			5(11.9%)	37(88.1%)		
Poor	60	4(6.7%)	56(93.3%)			2(3.3%)	58(96.7%)		
Lymph Node Metastasis									
N0 + N1	73	29(39.7%)	44(60.3%)	10.11	*p* < 0.005	13(17.8%)	60(82.2%)	5.62	*p* < 0.025
N2 + N3	57	7(12.3%)	50(87.7%)			4(7.0%)	53(93.0%)		

Part II: Correlation between RBP2 and Snail-1 expression in primary gastric cancer specimen									
		RBP2 expression			
Snail-1 expression	Total	0––1	2––3	χ^2^ value	*p* value
0––1	17	11(64.7%)	6(35.3%)	14.75	*p* < 0.005
2––3	113	25(22.1%)	88(77.9%)		

χ^2^ tests were used

0–1 poor gene expression; 2–3 strong gene expression

### RBP2 plays a key role in GC progression and helps to maintain the stemness of gastric cancer cells

Our previous studies have evidenced the critical role of RBP2 in the development of GC and HCC [21, 22], however whether it appears to be essential in malignant progression of human tumors has not been fully investigated. For this, we depleted RBP2 expression in GC cell lines (Supplementary Figure 1b) since our preceding data evidenced its high expression both in GC tissues and cell lines [21]. After suppression of RBP2, the capacity of invasion and migration of GC cells was significantly inhibited (Figure 1c and 1d). Consistent with this, wound-healing assay confirmed the decreased mobility of GC cells with RBP2 depletion (Supplementary Figure 1c). In addition, we depleted RBP2 expression in undifferentiated gastric cancer cells (HGC-27) which exhibit spindle-, fibroblast-like morphology. After RBP2 was inhibited with siRNA, HGC-27 cells underwent switch of spindle-, fibroblast-like appearance to cobblestone-like morphology (Supplementary Figure 1d), suggesting its key role in the maintenance mesenchymal phenotype. Some data have proved that tumor cells often obtain the stem cell property during EMT which is the reason why it is sometimes easy for cancer to relapse [4], thereby next we tried to determine the role of RBP2 in stem cell property maintenance. Accordingly, RBP2 inhibition evidently decreased anchorage-independent proliferation of GC cell lines as shown by the diminished ability to form spheroid colonies (Figure 1e). Altogether, these data demonstrated the critical role RBP2 played in GC cell invasion and migration, as well as in the maintenance of stem cell property *in vitro*. In the following experiments, we performed nude mice tail vein injection of GC cells *in vivo*. Firstly, we constructed BGC-823 cell lines with stable-transfection of RBP2 shRNA using lenti-virus. These cells expressed sustained lower RBP2 protein as compared with matched control (Figure 1g). We injected these cells into nude mice via tail vein with 400,000 cells per mouse and sacrificed the mice to harvest organs that contained metastatic tumor nodules 1 month later. Mice livers and kidneys suffered from tumor metastasis the most and 100% and 89% mice in the control group had liver and kidney metastasis respectively (Figure 1f and 1h, Supplementary Figure 1e and 1f). In marked contrast, only 30% and 20% mice had obvious liver and kidney metastasis respectively in RBP2 shRNA group (Figure 1h and Supplementary Figure 1f), suggesting that RBP2 inhibition decreased tumor metastasis *in vivo*, which was in agreement with the previous results *in vitro*. Moreover, we performed HE staining in slides containing tissues of liver, gallbladder, lung, kidney and brain from mice and found the reduced number of metastatic nodules in all the above organs in RBP2 shRNA group (Figure 1i and 1j, Supplementary Figure 1g), forcefully confirming the results *in vitro*. Furthermore, we also overexpressed RBP2 in gastric cancer cell lines and found their enhanced ability to migrate (Figure 1k).

### RBP2 regulates both EMT-related and stemness-related genes expression

**Figure 2:**
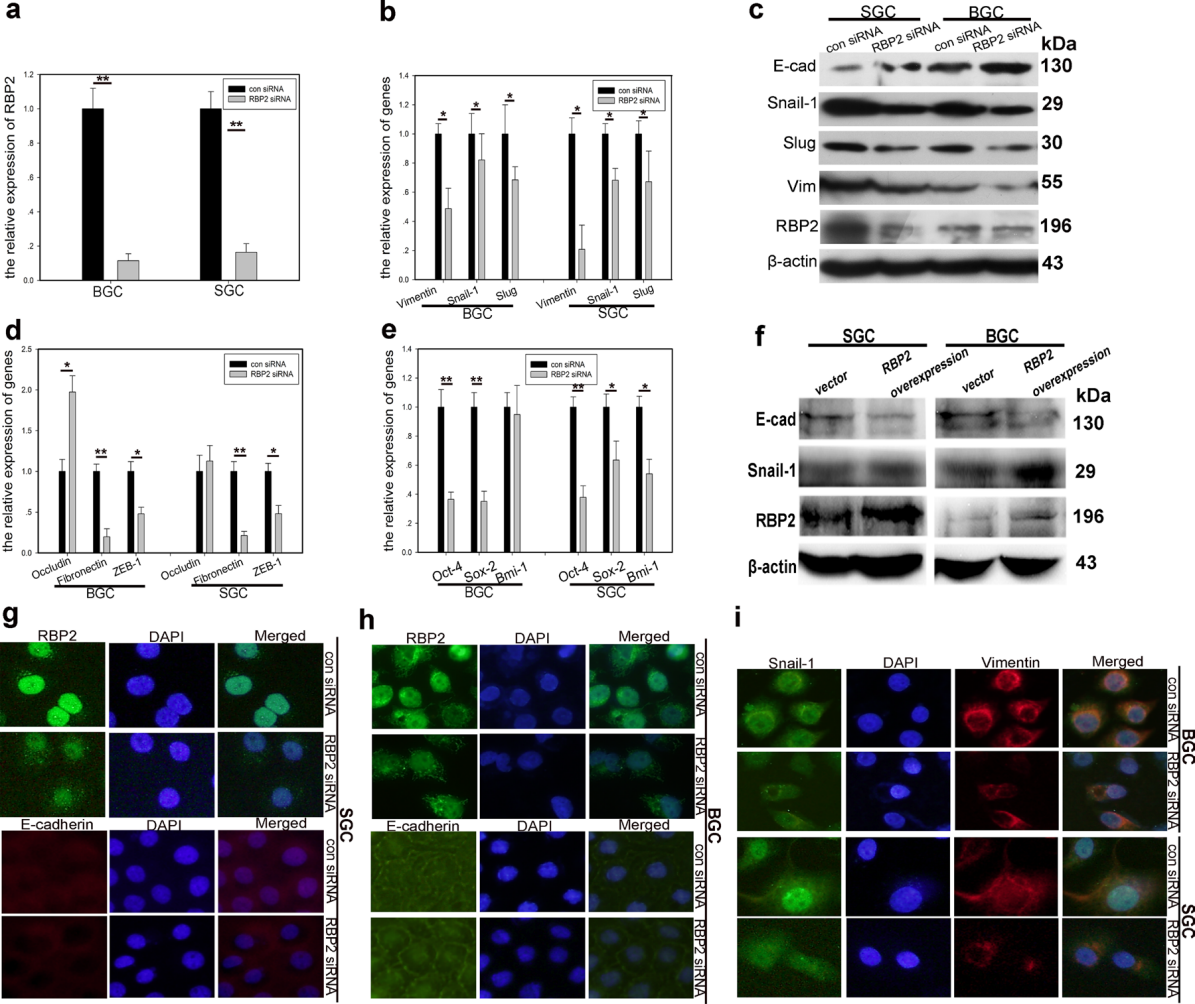
RBP2 regulates the expression of EMT-related and stemness-related genes **a.** RBP2 mRNA is significantly inhibited with siRNA treatment in GC cells using QRT-PCR. Data are mean ± SD of 3 biological replicates, **p < 0.01 compared with negative control. **b.** mRNA levels of EMT-related, mesenchymal genes were decreased with RBP2 suppression using QRT-PCR. Data are mean±SD of 3 biological replicates, *p < 0.05 compared with negative control. **c.** Upregulation of epithelial marker (E-cadherin) and downregulation of mesenchymal markers (Snail-1, Slug and Vimentin) with RBP2 inhibition using western blot. Representative images are shown here from three independent biological replicates. **d** and **e.** QRT-PCR shows upregulation of epithelial marker (Occludin) and downregulation of mesenchymal markers (Fibronectin and ZEB-1) and stemness related genes (Sox2, Oct4 and Bmi-1). Data are mean ± SD of 3 biological replicates, * and **p < 0.05 and < 0.01 compared with negative control. **f.** RBP2 overexpression enhanced mesenchymal genes expression while decreased E-cadherin expression with western blot assay. **g–i.** g and h Upregualtion of E-cadherin with RBP2 knockdown in BGC-823 cells and SGC-7901 cells respectively using immunofluorescence. **i** Expression of mesenchymal markers (Snail-1 and Vimentin) is decreased when RBP2 is downregulated using immunofluorescence. Representative images are shown herefrom three independent biological replicates. Original magnification, × 100.

To this end, we had known that RBP2 was critical for cell invasion and migration and tumor metastasis *in vitro* and *in vivo* respectively, but the underlying mechanism was unknown. It is well established that EMT is characterized by downregulation of epithelial markers (e.g. E-cadherin and Occludin) and upregulation of mesenchymal markers (e.g. Vimentin, Snail-1, Slug, Twist and ZEB1) [1–3], therefore we tested these markers in the following experiments. Accordingly, Vimentin, Snail-1 and Slug expression was downregulated and E-cadherin was upregulated with RBP2 suppression in GC cell lines as determined with QRT-PCR and western blot methods respectively (Figure 2a, 2b and 2c, Supplementary Figure 1h and 1j). Other epithelial markers, such as Occludin, and mesenchymal markers, such as Fibronectin and ZEB-1, were increased and decreased respectively with RBP2 inhibition (Figure 2d). We also tested the stemness-related genes expression after RBP2 depletion and found their suppression (Figure 2e, Supplementary Figure 1i), in line with the changes of mesenchymal markers. Reversely, when RBP2 was overexpressed in gastric cancer cell lines, Snail-1 protein increased whereas E-cadherin decreased, confirming the role of RBP2 in the regulation of EMT-related genes expression (Figure 2f). To further testify the regulation of EMT-related genes by RBP2, we performed immunofluorescence assay and found the same results as previously displayed (Figure 2g, 2h and 2i). Thus, in conclusion, RBP2 participated in EMT process and stem cell property maintenance by regulating EMT-related and stem cell property related genes respectively.

### RBP2 can be induced by TGF-β1 relying on Smad3 phosphorylation

**Figure 3:**
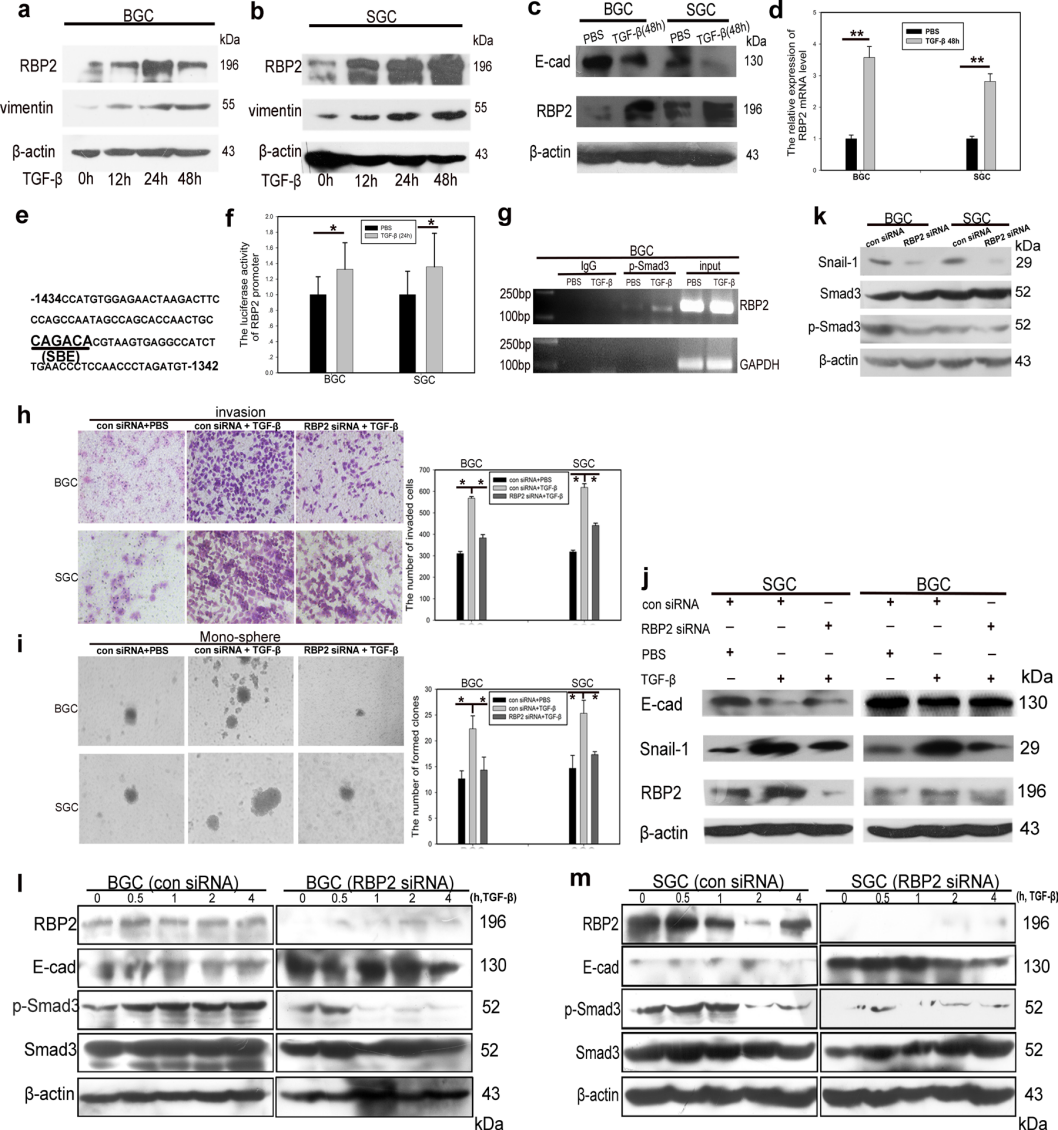
RBP2 can be induced by TGF-β1 and RBP2 is essential for EMT induced by TGF-β1 **a.** and **b.** TGF-β1 induces RBP2 protein expression in a time dependent manner in BGC-823 and SGC-7901 cells respectively. Representative images are shown here from three independent biological replicates. **c.** Upregulation of RBP2 and downregulation of E-cadherin by TGF-β1 treatment for 48 hours using western blot. Representative images are shown here from three independent biological replicates. **d.** QRT-PCR shows induction of RBP2 mRNA level in GC cell lines by TGF-β1 treatment for 48 hours. Data are mean ± SD of 3 biological replicates, **p < 0.01 compared with negative control. **e.** SBE element in RBP2 promoter. **f.** Increase of promoter luciferase activity of RBP2 by TGF-β1 treatment for 12 hours. Data are mean ± SD of 3 biological replicates, *p < 0.05 compared with negative control. **g.** p-Smad3 directly binds to RBP2 promoter using ChIP assay. **h.** and **i.** Invasion and maintenance of stem cell property of GC cells induced by TGF-β1 can be abrogated with RBP2 inhibition respectively. Data are mean±SD of 3 biological replicates, *p < 0.05 compared with negative control. Original magnification, × 60 and × 40 respectively. **j.** RBP2 suppression reverted induction of mesenchymal markers and inhibition of epithelial marker mediated by TGF-β1. Representative images are shown here from three independent biological replicates. **k.** Inhibition of RBP2 decreases endogenous Smad3 phosphorylation. Representative images are shown here from three independent biological replicates. **l.** and **m.** Pre-treatment with RBP2 siRNA in GC cell lines markedly retrieves Smad3 activation (formation of p-Smad3) induced by TGF-β1 in different time points in BGC-823 and SGC-7901 cells respectively.

Diverse factors give rise to EMT which, in a large part, fosters tumor metastasis [24, 25]. TGF-β1 is one of the best-known inducers [26]. A number of downstream target proteins can be activated upon TGF-β1 treatment. However, whether some key epigenetic molecules can be induced by TGF-β1 and the underlying mechanism remain unclear. Here we focus on RBP2 because previous work have shown it may be closely associated with some pivotal signaling pathways, including TGF-β1 [23]. Our previous results also indicated its critical role in EMT (Figures 1 and 2). Therefore, at this point, we want to know how TGF-β1 can have effect on RBP2. For this, we detected RBP2 expression in GC cell lines (BGC-823 and SGC-7901) undergoing TGF-β1 (5ng/ml) treatment. Surprisingly, RBP2 can be induced by TGF-β1 in a time dependent manner (Figure 3a and 3b), similar to the the change of the known downstream target vimentin (Figure 3a and 3b). To verify the effect of TGF-β1 treatment, we observed cell morphology change and found epithelial cells undergoing switch of cobblestone-like appearance to a spindle-, fibroblast-like morphology after TGF-β1 addition for 48 hours, but this phenomenon vanished after deprive of it 24 hours later (Supplementary Figure 1k), suggesting the above change was TGF-β1 dependent. In addition, E-cadherin, one of the epithelial markers, was blunted after 48 hours upon TGF-β1 treatment (Figure 3c), further confirming effectiveness of the inducer. At the same time, both mRNA and protein levels of RBP2 overtly increased when GC cells were subjected to TGF-β1 treatment for 48 hours (Figure 3c and 3d). Next we sought to find the underlying mechanism for RBP2 induction. Luckily, we found the conserved SBE (Smad3 binding sequence, CAGACA) [27, 28] in RBP2 promoter, locating on upstream of TSS (−1382 to −1387, Figure 3e) (UCSC, **http://genome.ucsc.edu/**). At this point, we constructed RBP2 promoter plasmid containing SBE and transfected it into GC cell lines. 24 hours later, we found the significant enhancement of RBP2 promoter activity upon TGF-β1 treatment (Figure 3f). ChIP assay confirmed phosphorylated Smad3 bound directly to RBP2 promoter via SBE recognition as displayed in Figure 3g. Phosphorylated Smad3 connected TGF-β1 treatment with RBP2 induction and this suggested that some epigenetic molecules may participate in key biological process induced by TGF-β1.

### RBP2 is crucial for EMT induced by TGF-β1 in GC

Our previous data had confirmed that RBP2 can be induced by TGF-β1 and, to this end, we sought to validate if RBP2 was crucial for EMT induced by TGF-β1. For this, we pre-treated GC cell lines with RBP2 siRNA and then added TGF-β1 into them. Strikingly, the enhancement of cell invasion as well as anchorage-independent proliferation of GC cells was abrogated by RBP2 siRNA pre-treatment (Figure 3h and 3i), which suggested that RBP2 may be crucial for EMT and stem cell property maintenance induced by TGF-β1. To further validate this, we determined EMT-related genes expression using western blot in the above settings. As expected, RBP2 siRNA pre-treatment which decreased RBP2 expression reversed, at least in part, the induction of mesenchymal markers (Vimentin, Snail-1 and Slug) and downregulation of epithelial markers (E-cadherin) upon TGF-β1 treatment (Figure 3j). Although the above results indicated the crucial role of RBP2 in EMT process induced by TGF-β1, the underlying mechanism needed to be unveiled. For this, we measured the endogenous p-Smad3 change after RBP2 depletion since EMT-related genes regulation by TGF-β1 is, in most cases, p-Smad3 dependent. As exhibited in Figure 3k, RBP2 suppression notably inhibited endogenous Smad3 phosphorylation. However what is the case upon TGF-β1 activation ? Pre-treatment with RBP2 siRNA in GC cell lines markedly retrieved Smad3 activation (formation of p-Smad3) induced by TGF-β1 in different time points (Figure 3l and 3m). Immunofluorescence assay corroborated the decreased nuclear translocation of Smad3 induced by TGF-β1 with RBP2 siRNA pre-treatment (Supplementary Figure 2a). Therefore there was a mutual regulation between Smad3 and RBP2 which intrigued our interest to uncover the underlying mechanism. It is worth noting that E-cadherin, one of the most important epithelial markers, consistently displayed inverse relationship with RBP2 (Figure 2c, 2f, 2g and 2h, Figure 3c, 3i and 3m), which may implicate RBP2 exerted regulation effect on it during EMT process.

### RBP2 participates in the regulation of GC progression by directly suppressing E-cadherin expression

**Figure 4:**
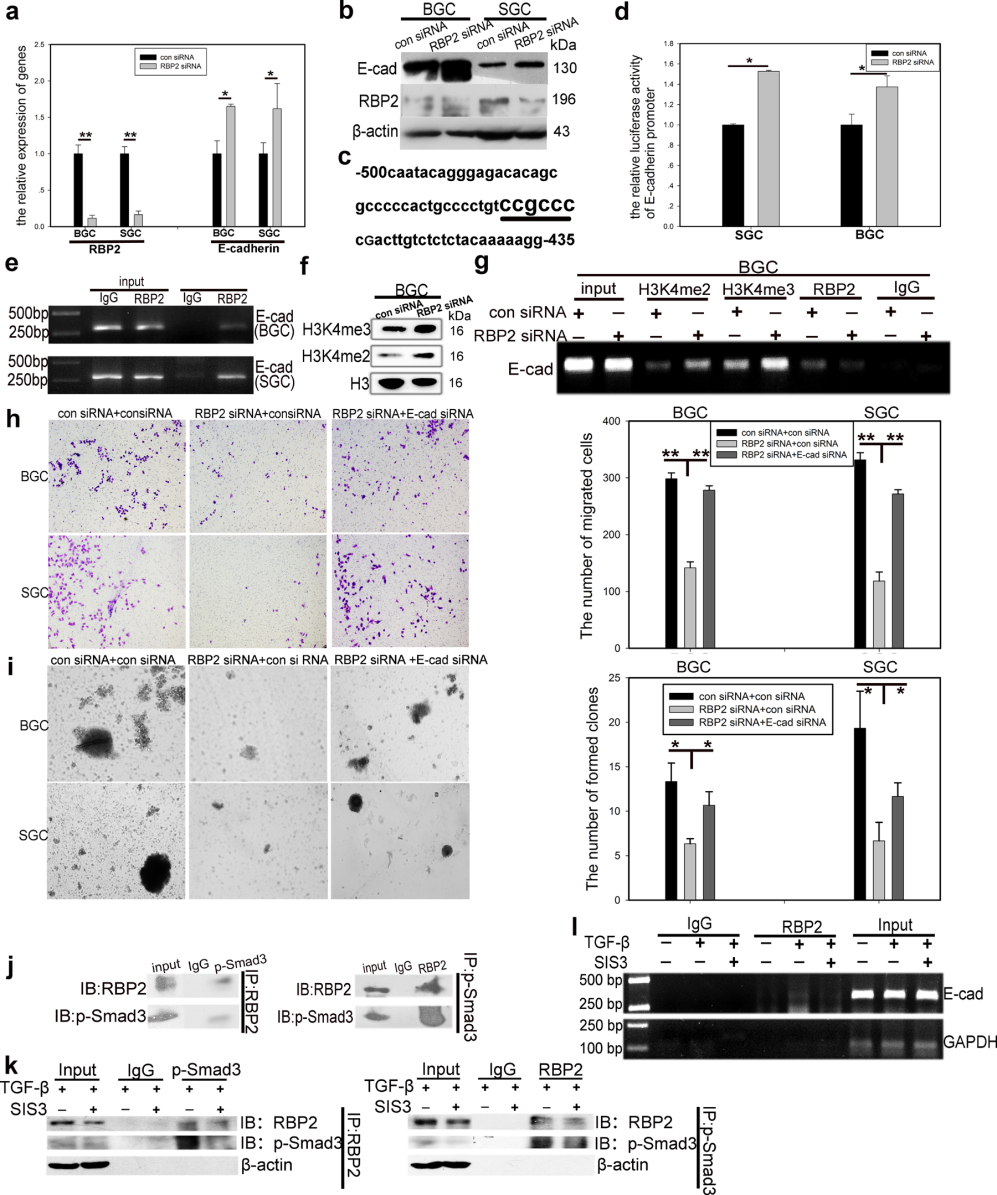
RBP2 participates in the regulation of GC progression by directly suppressing E-cadherin expression and p-smad3 binds to RBP2 and recruits it to the promoter of E-cadherin to reinforce suppression effect **a.** and **b.** QRT-PCR and western blot results show downregulation of RBP2 and upregulation of E-cadherin in GC cells with RBP2 inhibition. Data are mean ± SD of 3 biological replicates, * and **p < 0.05 and < 0.01 compared with negative control. **c.** RBP2 recognizing element CCGCCC in E-cadherin promoter. **d.** E-cadherin promoter luciferase activity increases when RBP2 is depleted in GC cells. Data are mean ± SD of 3 biological replicates, *p < 0.05 compared with negative control. **e.** ChIP assay shows direct binding of RBP2 to E-cadherin promoter. **f.** The levels of H3K4me2 and H3K4me3 increase in GC cells with RBP2 inhibition. **g.** Binding of RBP2 to E-cadherin promoter is its histone demethylase dependent. **h.** and **i.** Decrease of migration and maintenance of stem cell property of GC cells induced by RBP2 inhibition is abrogated by E-cadherin suppression simultaneously. Data are mean±SD of 3 biological replicates, * and **p < 0.05 and < 0.01 compared with negative control. Original magnification, × 40. **j.** Immunoprecipitate assay indicates the interaction between p-smad3 and RBP2. Representative images are shown here from three independent biological replicates. **k.** Immunoprecipitate assay demonstrates SIS3 decreases RBP2 induction by TGF-β1 and diminishes the interaction between p-smad3 and RBP2. **l.** Binding of RBP2 to E-cadherin promoter decreases with SIS3 treatment.

In the next step, we focused on the potential regulation of E-cadherin by RBP2. First we measured E-cadherin RNA and protein expression after RBP2 depletion in GC cell lines. As shown in Figure 4a and 4b, E-cadherin was overtly upregulated both at RNA and protein level respectively with RBP2 suppression. Next, we constructed E-cadherin promoter plasmid containing conserved RBP2 recognizing sequence (CCGCCC) [29], located in upstream of TSS (−459 to −464, Figure 4c) (UCSC, http://genome.ucsc.edu/). E-cadherin promoter activity was notably enhanced with RBP2 inhibition as indicated by the increased luciferase activity shown in Figure 4d. EMSA and ChIP assays in GC cell lines indicated that RBP2 can directly bind to E-cadherin promoter in the region that contains conserved RBP2 recognizing sequence (CCGCCC) (Figure 4e and Supplementary Figure 2c). Using IHC staning, we found the negative correlation between RBP2 and E-cadherin expression in GC tissues (Supplementary Figure 2b). To be specific, RBP2 expression was strong in the regions where E-cadherin was lost whereas the regions (relative normal epithelial cells) exhibiting obvious membrane expression of E-cadherin failed to have nuclear RBP2 expression. RBP2 is a kind of demethylasse which belongs to a family of demethylase, specific for tri- and dimethylated lysine 4 on histone 3 [19]. Thus we wanted to know whether RBP2 regulating E-cadherin was its histone demethylase activity dependent. In BGC-823 cells, RBP2 depletion remarkably enhanced H3K4me2 and H3K4me3 expression (Figure 4f). At the same time, H3K4me2 and H3K4me3 bound to E-cadherin promoter significantly increased after RBP2 suppression (Figure 4g), which may suggest that suppression of E-cadherin expression by RBP2 was its histone demethylase activity dependent and this needs further investigation. To determine whether RBP2 was critical for EMT and stemness maintenance through repressing E-cadherin, we treated GC cell lines with both RBP2 siRNA and E-cadherin siRNA and found that the capacity of cell migration and stemness maintenance inhibition by RBP2 knockdown were relieved by E-cadherin suppression simultaneously (Figure 4h and 4i), which potently implicated RBP2 played a pivotal role in EMT process by means of inhibiting E-cadherin expression. Furthermore, we also found that E-cadherin block can also abrogate the repression of vimentin and Snail-1 mediated by RBP2 knockdown (Supplementary Figure 2d and 2e), which may be accountable for the abrogation of capacity of cell migration and stemness maintenance inhibition by RBP2 knockdown in GC cell lines.

### p-smad3 binds to RBP2 and recruits it to the promoter of E-cadherin to reinforce suppression effect

Some data indicated that the nuclear Smad3 (p-Smad3) can form complexes to bind to E-cadherin promoter [30]. Combined with our previous result that RBP2 bound directly to E-cadherin promoter via recognizing the conserved CCGCCC elements, it was tempting to speculate that p-Smad3 may interact with RBP2 to form a complex to affect E-cadherin promoter activity. To this end, we performed immunoprecipitate assay and found the endogenous interaction between p-Smad3 and RBP2 (Figure 4j). To further confirm that this kind of interaction was p-Smad3 dependent, we employed SIS3, a specific p-Smad3 inhibitor. Of note, pre-treatment of SIS3 abrogated the induction of RBP2 by TGF-β1 and markedly decreased p-Smad3 formation (Figure 4k), the result of which was that the interaction between p-Smad3 and RBP2 was strongly blunted. Some histone demethylases can be recruited by p-Smad3 to the promoters of target genes [31], therefore it was conceivable that the H3K4 demethylase, RBP2, can also be recruited by p-Smad3 to the promoter of E-cadherin. To test this, we pre-treated BGC-823 cells with SIS3 and then suffered them from TGF-β1 addition. As expected, SIS3 remarkably attenuated the enhance of binding of RBP2 to E-cadherin promoter induced by TGF-β1 (Figure 4l). Thus p-Smad3 exerted dichotomous effects on the regulation of E-cadhein, on one hand, it bound to RBP2 promoter to increase its expression and the product repressed E-cadhein; on the other side, it can directly interacted with RBP2 and recruited it to E-cadhein promoter to enhance the suppression effect.

### E-cadherin exerts feedback regulation on RBP2 expression by inhibiting Smad3 phosphorylation

**Figure 5:**
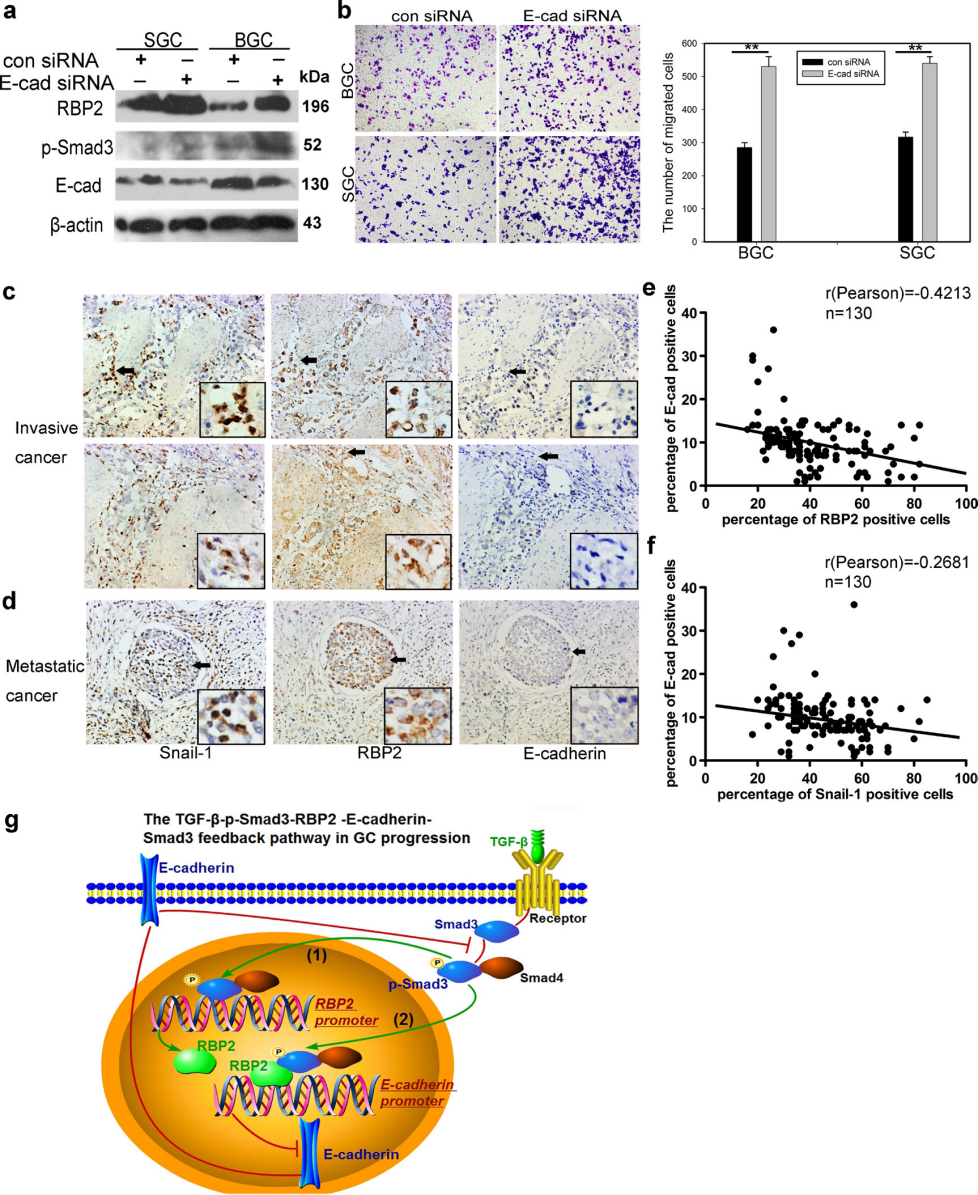
E-cadherin exerts feedback regulation on RBP2 expression by inhibiting Smad3 phosphorylation; Co-expression of RBP2 and Snail-1 and their inverse relationship with E-cadherin expression in primary gastric cancer tissues *in vivo* **a.** E-cadherin depletion upregulates the expression of p-smad3 and thus promotes RBP2 induction. Representative images are shown here from three independent biological replicates. **b.** E-cadherin knockdown enhances GC cell migration, similar to the effect of RBP2 induction. Data are mean±SD of 3 biological replicates, **p < 0.01 compared with negative control. Original magnification, × 40. **c. and d.** Co-expression of RBP2 and Snail-1 in invasive and metastatic gastric cancer tissues respectively where E-cadherin expression is lost. Representative images are shown here. Original magnification, × 40 (enlarged × 4). **e.** Inverse correlation between RBP2 and E-cadherin expression in the above tissues. **f.** Inverse correlation between Snail-1 and E-cadherin expression in the above tissues. **g.** Summary of the main positive feedback regulation circuit delineated in this paper.

As a key downstream target of both RBP2 and TGF-β1-(p-Smad3) signaling pathway, E-cadherin may have potential unrevealed function in EMT process. Cho IJ and Kim YW reported that E-cadherin antagonizes transforming growth factor β1 gene induction in hepatic stellate cells by inhibiting RhoA-dependent Smad3 phosphorylation [32], thereby we did the same in GC cell lines and found that depletion of E-cadherin indeed increased p-Smad3 expression, and thus the upregulation of RBP2 (Figure 5a), which may be because E-cadherin suppression abrogated the inhibition of RhoA-dependent Smad3 phosphorylation. Furthermore, E-cadherin suppression notably increased the capacity of migration of GC cells, similar to the effect of RBP2 induction (Figure 5b). Taken together, TGF-β1-(p-Smad3) signaling pathway induced RBP2 expression, resulting in E-cadherin repression, which promoted p-Smad3 formation, leading to more RBP2 induction. Therefore these results unveiled a novel TGF-β1-(p-Smad3)-RBP2-E-cadherin-Smad3 positive feedback regulation circuit during EMT and GC metastasis.

### Confirm co-expression of RBP2 and Snail-1 and their inverse relationship with E-cadherin expression in invasive and metastatic gastric cancer tissues *in vivo*

At last, we detected E-cadherin expression in the former mentioned tissues using IHC staining. Combined with the data in Result 1, we found co-localization or co-expression of RBP2 and Snail-1 in invasive (muscular invasion) and metastatic (metastatic foci in lymph duct) GC tissues which had no or very low E-cadherin expression (Figure 5c and 5d, Supplementary Figure 2f). In addition, we also detected TGFβRI which mediated the phosphorylation of Smad3 and was required for RBP2 upregulation in response to TGF-β1 in gastric cancer specimen. We found no significant difference of TGFβRI expression among different cancer differentiation state and pathological grades (data not shown). However, we indeed found co-expression of RBP2, Snail-1 and TGFβRI in some specimen (Supplementary Figure 2f). Both RBP2 and Snail-1 exhibited inverse relationship with E-cadherin (Figure 5e and 5f), further validating our preceding results that E-cadherin was negatively regulated by RBP2 *in vitro* (Figure 3c, Figure 4a and 4b).

## DISCUSSION

GC progression is a complicated process that involves a variety of factors [34], and the mechanism by which epigenetic molecules participate in it has yet to be fully uncovered. Here we present data demonstrating that the H3K4 demethylase, RBP2, plays a key role in promoting GC malignant progression. RBP2 can be transcriptionally induced by p-Smad3 upon TGF-β1 activation and it directly suppresses E-cadherin expression. Depletion of E-cadherin attenuates the inhibition of Smad3 phosphorylation, resulting in further RBP2 induction and thus constituting the TGF-β1-(p-Smad3)-RBP2-E-cadherin-Smad3 positive feedback regulation circuit that promotes GC malignant progression.

As a canonical signaling pathway in development of organs of vertebrates, TGF-β1 has dual roles in carcinogenesis, acting as a tumor suppressor in normal epithelial cells and in the early stages of tumorigenesis whereas serving as a tumor promoter during malignant progression [35, 36]. Exclusively, TGF-β1 is closely associated with EMT and contributes, predominantly, to distant metastatic dissemination of tumors [8]. Smad3 phosphorylation is a key step during TGF-β1 signaling activation and many factors have an effect on this process [37]. Some well established E-cadherin repressors will be induced following Smad3 phosphorylation, promoting EMT [38]. Mounting data have emerged to indicate that epigenetic modification is an indispensable part during tumorigenesis and malignant deterioration [39]. In the present data, we are focusing on RBP2, a newly identified histone demethylase, which is overexpressed in GC and its inhibition triggers cell senescence [21]. Other data suggested the potential relationship between RBP2 and TGF-β1 [23]. In addition, RBP2 induction is one of the five major traits of drug-tolerant subpopulation (cancer stem cells) in melanoma [40]. And it is well recognized that tumor cells undergoing EMT obtain stem cell property simultaneously [4]. So combined with all the information above, it raises the tantalizing possibility that RBP2 is involved in EMT and stemness property maintenance during malignant progression. Here, we indeed validate the role RBP2 plays in EMT and establish its relationship with TGF-β1. However, RBP2 can function both as a transcriptional repressor, inhibiting expression of downstream targets, and as a transcriptional enhancer, enhancing target genes expression, the accurate mechanism underlying this remains unclear. So in the following projects, we will pay more attention to the elaborate regulation of target genes mediated by RBP2.

Accumulating data implicate feedback regulation circuits exist universally in the pathogenic process [41–43], especially in tumorigenesis. It was reported that STAT3 activation of miR-21 and miR-181b-1 via PTEN and CYLD imposed feedback regulation on IL-6, which was in the upstream of STAT3 [44], and others published that Oct4 promoted the self-renewal and survival of embryonal carcinoma cells by feedback regulating AKT, which was in the upstream of Oct4 [45]. Basically, signaling cascade pathways can be inactivated by intrinsic factors to restrict overresponse to diverse stimulus, maintaining an internal homeostasis in the physiological state [46, 47]. However, once pathogenic changes take place, aberrant signaling cascade pathways can be sustainedly activated, forming feedback regulation circuits that lead to deterioration of diseases, including cancer. In the present work, we find a positive feedback regulation circuit in which RBP2 links TGF-β1 signaling activation to E-cadherin suppression and the latter gives rise to more RBP2 induction. RBP2 induction is a key step in this circuit and the dysregulation of RBP2 may jeopardize the homeostasis, fostering EMT and tumor distant dissemination. Thus more investigations are needed to delineate the mechanism for the formation of aberrant pathogenic, often tumorigenic, feedback regulation circuits to seek for more efficient strategy for the prevention and treatment of human diseases.

In summary, our present data illustrate RBP2 promotes GC malignant progression via directly repressing E-cadherin and this helps to form the pro-metastatic TGF-β1-(p-Smad3)-RBP2-E-cadherin-Smad3 positive feedback regulation circuit (Figure 5g). Therefore, targeting RBP2 may have therapeutic advantages for the prevention of tumor distant metastasis.

## MATERIALS AND METHODS

### Clinical samples

130 samples of progressive GC were got from patients. The specimen were collected immediately after surgery and stored at formalin. The diagnosis of GC for all patients was confirmed by histological examination.

### Animal experiment (*in vivo* experimental metastasis)

19 (7 weeks old) male nude mice were purchased from QING ZI LAN Animal Company (Nanjing, China) and divided into 2 groups. And then the mice were injected into 4 × 10^5^ cells per mouse through tail vein. One group were injected into RBP2 shRNA stable-transduction cells and the other group were injected into the matched control cells. One month later, the mice were sacrificed and the organs were harvested and photographed. Tissue sections were attained with traditional method and HE staining was performed.

### Statistical analysis

Quantitative data we got from experiments with biological replicates were shown as means (±SD or SEM). Student's *t* tests, χ2 tests and Pearson correlation efficiency analysis were used to analyze the differences between groups. Pearson correlation efficiency analysis was also used. *p* < 0.05 was considered to be statistically significant.

Other Methods can be found in Supplementary Methods.

## SUPPLEMENTARY DATA FIGURES



## Abbreviations

RBP2: retinoblastoma binding protein 2
GC: gastric cancer
EMT: epithelial-to-mesenchymal transition
TGF-β1: transforming growth factor β1
QRT-PCR: Quantitative real-time polymerase chain reaction siRNA; small interference RNA
shRNA: short hairpin RNA
IHC: immunohistochemistry
ChIP: chromatin immunoprecipitation
IP: immunoprecipitation
