# Supplementary material for: Histone demethylase RBP2 promotes malignant progression of gastric cancer through TGF-β1-(p-Smad3)-RBP2-E-cadherin-Smad3 feedback circuit
Source: Oncotarget. 2015 May 7;6(19):17661–74. doi: 10.18632/oncotarget.3756 (PMC4627336; doi:10.18632/oncotarget.3756)
Supplement: Supplementary file 1 [file oncotarget-06-17661-s001.pdf]

SUPPLEMENTARY DATA

RNA extraction, RT-PCR and real-time PCR

Cells were lysed by TRIzol reagent (invitrogen, USA) and the total RNA was extracted according to the protocol provided. And then the extracted RNA was reverse-transcribed with RevertAid First Strand DNA Synthesis (RT) kit (Fermentas, life science, Canada). Semi-quantitative PCR was performed using the previously got cDNAs as the templates. Real-time PCR was performed using SYBR Green kit (Takara, Japan) according to the protocol. The primers used in these experiments were the following:

RBP2:	
5-GCTGCTGCAGCCAAAGTTG-3	(forward)
5-AGCATCTGCTAACTGGTC-3	(reverse)
Snail-1	
5-CCCCAATCGGAAGCCTAACT-3	(forward)
5-GGTCGTAGGGCTG CTGGAA-3	(reverse)
ZEB-1:	
5-CACACTCTGGGTCTTATTCTCAAC-3	(forward)
5-CTCTTCTACCTCTTCTTCTTCCACTT-3	(reverse)
Sox-2:	
5-CGAGTGGAAACTTTTGTCGGA-3	(forward)
5-TGTGCAGCGCTCGCAG-3	(reverse)
E-cadherin:	
5-CCTTAGAGGTGGGTGACTACAA-3	(forward)
5-TCAGACTAGCAGCTTCGGAAC-3	(reverse)
Occludin:	
5-AGTGGTTCAGGAGCTTCCATTA-3	(forward)
5-TATTCATCAGCAGCAGCCATGT-3	(reverse)
Fibronectin:	
5-GGTGACACTTATGAGCGTCCTAAA-3	(forward)
5-TACAATCTACCATCATCCAGCCT-3	(reverse)
Slug:	
5-CTGCGGCAAGGCGTT-3	(forward)
5-GCAGTGAGGGCAAGAAAAAGG-3	(reverse)
Oct-4:	
5-ACCGAGTGAGAGGCAACC-3	(forward)
5-TGAGAAAGGAGACCCAGCAG-3	(reverse)
Bmi-1:	
5-AAATGCTGGAGAACTGGAAAG-3	(forward)

5-CTGTGGATGAGGAGACTGC-3	(reverse)
Vimentin:	
5-AAAACACCCTGCAATCTTTCAGA-3	(forward)
5-CACTTTGCGTTCAAGGTCAAGAC-3	(reverse)
β-actin:	
5-AGTTGCGTTACACCCTTTCTTG-3	(forward)
5-CACCTTCACCGTTCCAGTTTT-3	(reverse)

Cell culture and siRNA interference

BGC-823 and SGC-7901 cells were cultured in RPMI-1640 medium (Gibco, USA) mixed with 10% fetal bovine serum (Gibco, USA) in 5% CO_2 atmosphere at 37°C. RBP2 siRNA was purchased from invitrogen (USA). And E-cadherin siRNA was got from GenePharma Company (Shanghai, China). The sequences for RBP2 siRNA and its negative control siRNA were 5′- CCA GCA CCA CCU CCU UCC UUC AUA A -3′ and 5′- CCU ACA UCC CGA UCG AUG AUG UUG A -3′ respectively; the The sequences for E-cadherin siRNA and its negative control siRNA were 5′-CAG ACA AAG ACC AGG ACU A-3′ and 5′- CAA UAA UCC CGA UGC AUG AUG AAG A -3′ respectively. siRNAs were transfected into cells with Lipofectamine 2000 (Invitrogen, Carlsbad, CA, USA) according to the protocol.

Protein extraction and western blot

Protein lysis buffer was used to extract total proteins from cells according to the protocol and the extracted proteins were resolved on SDS-PAGE. After transferring membrane, blocking non-specific antigens and primary antibodies incubation overnight at 4°C, secondary antibodies were added. Antibody binding was detected by chemiluminescence (Millipore, USA) according to the protocol. The primary antibodies used in these experiments were the following: RBP2 (Abcam USA), β-actin (Sigma, USA), E-cadherin (Cell Signaling, USA), p-Smad3 (Cell Signaling, USA), Smad3 (Bioss, China), Snail-1 (Abcam USA), Slug (Abcam USA) and Vimentin (R&D, USA).

plasmid transfection and Luciferase reporter gene assay

RBP2 overexpression plasmid was transfected into GC cell lines using Roche Transfection Reagent (Roche, USA) according to the protocol. 48 hours later, cells were harvested and subjected to subsequent assays. PGL- RBP2 promoter plasmid or PGL-E-cadherin promoter plasmid, and PGL-TK were co-transfected into GC cells using Roche Transfection Reagent (Roche, USA) according to the protocol. The cells were lysed 24 or 48 hours later, mixed with the dual luciferase assay reagent (Promega, USA). Relative luciferase activity was calculated by normalizing the firefly luminescence as to the renilla luminescence.

TGF-β1 and SIS3 treatment

TGF-β1 was purchased from PeproTech, USA. The concentration used in the experiments was 5 ng/ml and the time after adding varied according to the experiments. SIS3 was got from BioVision (USA) and the concentration used in the experiments was 5 uM. SIS3 was added to the medium 12 hours before TGF-β1 treatment.

Cell invasion and migration

For transwell migration assay, collagen-coated Transwells were used to load 50000 GC cells per well for 48 hours and the chemoattractant used was the medium containing 20% FBS. Cells were calculated in 6 different fields with an inverted microscope. All experiments were performed in triplicate. For transwell invasion assay, Matrigel-coated Transwell chambers were used to load 50000 GC cells per well for 36 or 48 hours and the chemoattractant used was the medium containing 20% FBS. Cells were calculated in 6 different fields with an inverted microscope. All experiments were performed in triplicate.

Wound-healing

For the wound-healing assay, cells were seeded to confluence in a 6-well plate, and the cell surface was scratched using a pipette tip. And the cells were cultured in a serum-free 1640 medium to allow to repopulate the scratched area for 3 days. Accurate wounds measurements were taken at 0, 24, 48 and 72 hours to calculate the migration rate. The equation used in this experiment is the following: percentage wound healing = [(wound length at 0 hour) − (wound length at 24, 48 and 72 hours)]/(wound length at 0 hour) × 100.

Sphere formation

Sphere formation assays were performed with the traditional method. Single cells were plated at 10,000 cells/mL on 96-well ultra-low attachment plates

(Corning Life Sciences) in serum-free RPMI-1640/10 mM HEPES medium supplemented with 10 ng/mL bFGF (PeproTech, USA) and 20 ng/mL EGF (PeproTech, USA) and fresh medium was supplemented every 3 days. The spheroid colonies formed were calculated at day 14.

IHC

Paraffin-embedded tissue sections were got from GC patients. Sections were stained using a conventional immunohistochemistry procedure. To be brief, sections were deparaffinized and rehydrated. And then Antigen retrieval and endogenous peroxidase inactivity were performed using the traditional method. After that, sections were blocked with 10% goat serum and the corresponding primary antibodies were used to incubate overnight at 4°C. The primary antibodies used in this experiment were: RBP2 (Sigma, USA), E-cadherin (Cell Signaling, USA) Snail-1, (Abcam USA) and TGFβRI (Abcam USA). After incubation with secondary antibodies for 30 minutes, the antibody binding was detected by the avidin-biotin-peroxidase method with DAB staining (Vector Laboratories, Burlingame, CA, USA). Gene expression was scored as 0 (no visible staning in tumor cells), 1 (poor, nuclear or membrane positive staining in 0–30% tumors), 2 (moderate, nuclear or membrane positive staining in 31–60% tumor cells), 3 (strong, more than 60% nuclear or membrane positive staining in tumor cells) on the basis of the intensity and percentage of genes staining.

Immunofluorescence

Cells were seeded on a glass cover in a 6-well microtiter plate. Then the cells were fixed with methanol for 10 minutes. Cells were permeabilized by incubation with PBS 0.1% Triton X-100 (Sigma) for 5 minutes, and incubated with the corresponding primary antibodies overnight at 4°C. And then the cells would be stained with the corresponding secondary antibodies. Nuclei were counterstained with 40,6-diamidino-2-phenylindole (DAPI). Photographs were taken using an OLYMPUS fluorescence microscope.

EMSA

Nuclear extracts from GC cells were incubated with 4 ul Binding buffer, 1ul poly [d(I-C)] [1µg/µl], 1 ul poly L-lysine [0.1 µg/µl] and DIG-labeled probes (Roche, USA) of E-cadherin promoter (5′AATGTCCGCCCCCGCCCCCGCCCCGAC TTGTAA3′) in a 20 ul reaction volume. DIG Gel Shift Kit (Roche, USA) was used in this experiment. For super shift assay, the nuclear extracts were incubated with 400 ng of antibody. 6% polyacrylamide gel was used to electrophorese the samples and then they were transferred onto a nylon membrane and ultraviolet-crosslinked. CSPD (Roche, USA) chemiluminescence reaction was used to detect the DIG-labeled probe.

ChIP

ChIP assay kit (millipore, USA) was used to treat the prepared GC cells according to the protocol provided. The DNA samples we finally got were analysed with PCR method. The primes used in this experiment were the following: RBP2 promoter: 5′GAACTAAGACTTCCCA GCCAA3′(Forward); 5′GTGAGTCAAGTCCTTAAAAT AAATA3′(Reverse). E-cadherin promoter: 5′GAGAGTC TCTTGAACCCGGCAG3′(Forward); 5′TGTAGAGAGA CAAGTCGGGGCG3′(Reverse).

IP (Immunoprecipitation) assay

For immunoprecipitation, GC cells were cultured in 10-cm dishes and transfected with the appropriate siRNAs if needed. Pierce IP/Co-IP Kit (USA) was used in this experiment according to the protocol provided. To be specific, GC cell lysates were incubated with 2–5 µg of antibody overnight at 4°C on a rotator. After adding 25 µl of Protein A/G Magnetic Beads (Pierce, USA) for 1 hour at 4°C, we got The protein–antibody–Protein A/G Magnetic Beads complexes. The immunoprecipitated complexes were resuspended in reducing sample buffer after extensive washing with lysis/wash buffer and boiled for 15 minutes. Supernatants were got after centrifugation to pellet the Magnetic Beads and then were subjected to SDS-PAGE and immunoblotting.

Construction of lentiviral vectors

Stable knockdown BGC-823 cells were generated using short hairpin RNAs (shRNA) directed against human RBP2 gene constructed in pLV3 vector obtained from GenePharma (Shanghai, China). Control cells were created by using a plasmid carrying nontargeting control sequence.

Animal experiment (*in vivo* experimental metastasis)

19 (7 weeks old) male nude mice were purchased from QING ZI LAN Animal Company (Nanjing, China) and divided into 2 groups. And then the mice were injected into 4×10^5 cells per mouse through tail vein. One group were injected into RBP2 shRNA stable-transduction cells and the other group were injected into the matched control cells. One month later, the mice were sacrificed and the organs were harvested and photographed. Tissue sections were attained with traditional method and HE staining was performed.

SUPPLEMENTARY FIGURES

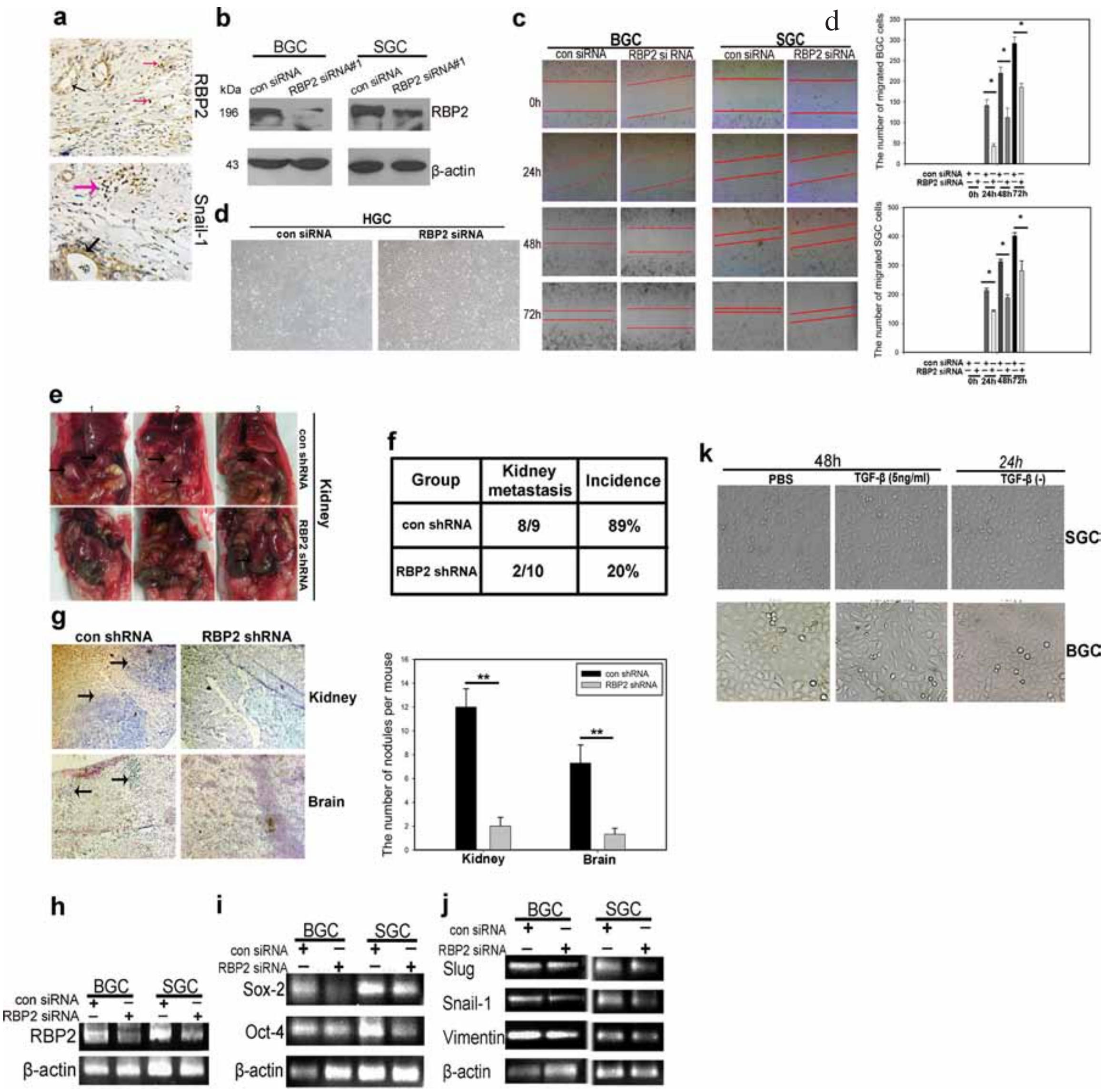

Supplementary Figure S1: a. RBP2 expression in regions with different differentiation status: Red arrows-poor differentiated regions; Black arrows-well or moderate differentiated regions. b. The expression of RBP2 was significantly inhibited with 2 kinds of specific RBP2 siRNAs. **c.** Wound-healing assay shows inhibition of RBP2 blunts cell migration. Data are mean ± SD of 3 biological replicates, $*p < 0.05$ compared with negative control. Original magnification, ×10. **d**. RBP2 depletion in HGC-27 cells resulted in the morphology change. **e**. RBP2 suppression significantly decreases kidney metastasis. Black arrows indicate the metastatic tumor nodules. **f**. Decrease of kidney metastasis incidence in RBP2 shRNA group. **g**. HE staining of kidney and brain, which shows decrease of metastatic tumor nodules formed in RBP2 shRNA group. Representative images are shown here. Black arrows indicate the metastatic tumor nodules. Original magnification, ×40. Data are mean ± SD of 3 biological replicates, $**p < 0.01$ compared with negative control. **h**. Semi-quantitative PCR detects RBP2 depletion with siRNAs treatment. Representative images are shown here from three independent biological replicates. **i**. Stemness related genes are downregulated when GC cell were treated by RBP2 siRNA with semi-quantitative PCR method. Representative images are shown here from three independent biological replicates. **j**. Mesenchymal genes expression with decreases RBP2 siRNA treatment using semi-quantitative PCR method. Representative images are shown here from three independent biological replicates. **k**. GC cells undergo changes from cobblestone-like appearance of epithelial cells to a spindle-, fibroblast-like morphology dependent on TGF-β1. Representative images are shown here from three independent biological replicates.

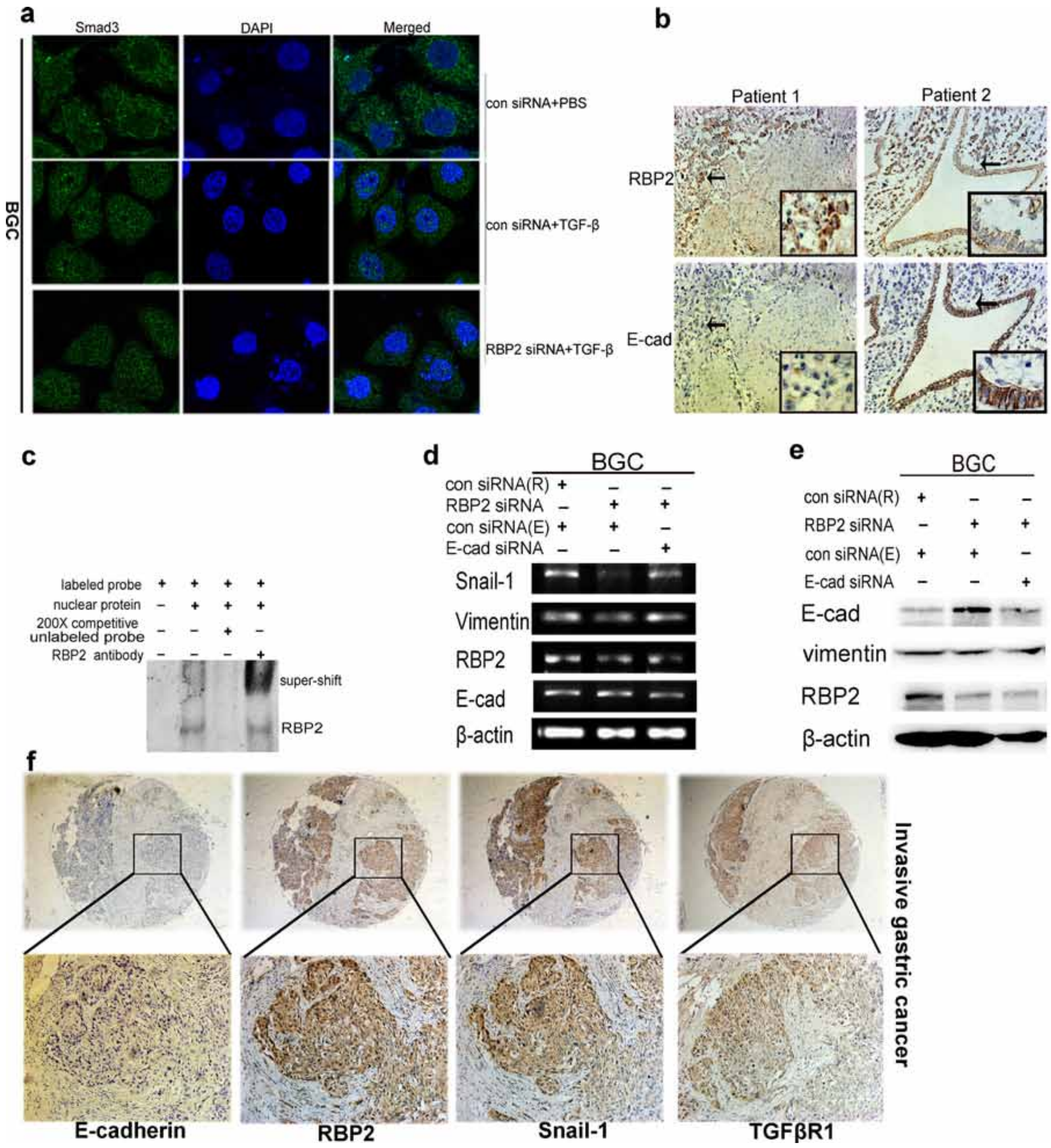

Supplementary Figure S2: a. Nuclear translocation of Smad3 induced by TGF-β1 decreases with RBP2 siRNA pre-treatment. Representative images are shown here**. b.** IHC staining of RBP2 and E-cadherin in GC tissues uncovers their inverse expression relationship. Representative images are shown here. Original magnification, ×40 (enlarged ×4). **c**. EMSA detected binding of RBP2 to CCGCCC element within E-cadherin promoter. **d**. and **e**. E-cadherin block abrogates the repression of vimentin and Snail-1 mediated by RBP2 knockdown using Semi-quantitative PCR and western blot respectively. Representative images are shown here from three independent biological replicates. **f**. IHC staining for E-cadherin, RBP2, Snail-1 and TGFβRI in invasive gastric cancer tissues. Original magnification, ×10 (enlarged ×4).
